# Draft genome sequence of *Pseudomonas aeruginosa* MLSE01 isolated from seawater-driven microbial fuel cell

**DOI:** 10.1128/mra.00198-26

**Published:** 2026-04-27

**Authors:** Shovon Shaha, Tanvir Ahmmed, Md. Ashikuzzaman Antor, Abu Reza, Borshon Karmoker Pranto, Sadiatul Keya, Mohammad Abu Hena Mostofa Jamal

**Affiliations:** 1 Department of Biotechnology and Genetic Engineering, Faculty of Biological Sciences, Islamic University 247297, Kushtia, Bangladesh; 2 Department of Immunology, BIRDEM General Hospital, Dhaka, Bangladesh; 3 Laboratory of Medical and Environmental Biotechnology, Islamic University 247297, Kushtia, Bangladesh; 4 Department of Genetic Engineering and Biotechnology, Faculty of Health and Life Sciences, Daffodil International University 130058, Dhaka, Bangladesh; University of Southern California, Los Angeles, California, USA

**Keywords:** microbial fuel cell, exoelectrogenic bacteria, genome, *Pseudomonas aeruginosa* MLSE01, genome analysis

## Abstract

The present study reports the draft of the entire genome sequence of *Pseudomonas aeruginosa* MLSE01, a strain isolated from the Kuakata seawater-driven microbial fuel cell in Bangladesh. The genome comprises a 6,332,769 bp chromosome assembled into 46 contigs, encoding 5,937 predicted genes.

## ANNOUNCEMENT

*Pseudomonas aeruginosa* is a gram-negative bacterium and is found in diverse locations, including soil, water, and the host body (1, 2). This study reports a draft genome sequence of *Pseudomonas aeruginosa* MLSE01, isolated from a seawater-driven microbial fuel cell used for electricity generation. The aim of genome analysis of this organism is to explore the genes associated to extracellular electron transfer, adaptability to diverse microbial fuel cell (MFC) conditions, and potential roles in xenobiotic degradation and bioenergy production.

A dual-chambered MFC was designed to detect the presence of exoelectrogenic bacteria in the seawater sample collected from Kuakata Sea Beach, Patuakhali, Bangladesh (located at 21.81372°N, 90.12240°E) (3). Approximately 15 mL of sandy water from about 10 cm below the sea surface was collected in a sterile falcon tube and transported immediately to the laboratory at 4°C. A salt bridge was used as the interconnecting proton exchange channel between the anode and the cathode chamber (4). The anode medium was prepared with the following components (g/L): glucose 2, NaCl 0.46, yeast extract 0.1, peptone 0.5, K_2_HPO_4_ 5.05, KH_2_PO_4_ 2.84, cysteine 0.025 (pH −7.1), and kept airtight using an anaerobic pack (Anaeropack). The cathode chamber was filled with 100 mM K_3_Fe(CN)_6_ solution in 100 mM KH_2_PO_4_ buffer (pH 7.0) and exposed to air. After constructing the MFC, 1 cm^3^ of seawater was inoculated into the anode, and the electricity generation by the exoelectrogenic bacteria was observed for 6 days. A loopful of anode biofilm from a fully functioning MFC was scratched and introduced on the “E-Z exoelectrogen growth and isolation medium” proposed by Nazeer et al., with some modifications, with the following components (g/L): glucose 2, NaCl 0.46 instead of NH_4_Cl, yeast extract 0.1, peptone 0.5, K_2_HPO_4_ 5.05, KH_2_PO_4_ 2.84, MnO_2_ 1.8, cysteine 0.025, and agar 15, and incubated at 37°C (5). Bacterial colonies decolorizing the MnO_2_ supplemented in the medium were taken and isolated on a fresh medium (5, 6). A single colony was grown in nutrient broth overnight at 37°C with 120 rpm agitation. One milliliter of culture was used for genomic DNA extraction using the Wizard HMW DNA Extraction following the manufacturer’s instructions (https://www.promega.com/-/media/files/resources/protocols/technical-manuals/500/wizard-hmw-dna-extraction-kit-protocol.pdf?rev=ee725be682a04f57a21438afa7debe51). After cell lysis, the debris was eliminated via centrifugation at 14,000 rpm for 5 min. Genomic DNA was purified from the supernatant using the Wizard Genomic DNA Purification Kit per manufacturer’s instructions (https://ita.promega.com//media/files/resources/protocols/technical-manuals/0/wizard-genomic-dna-purification-kitprotocol.pdf). NanoDrop spectrophotometer and Qubit 4.0 Fluorometer (1:200 Qubit Buffer ratio) were employed to evaluate the quality and concentration of the DNA (7). Genome sequencing was performed at the Genomic Center, ICDDR, B, Mohakhali, Dhaka-1212, Bangladesh. DNA fragments (200–300 bp) were size-selected with AMPure XP beads, libraries prepared with adapter ligation using Illumina DNA Prep Kit on an epMotion 5075, and sequenced on Illumina MiSeq with 2 × 150 bp paired-end reads. The given sequence was checked, trimmed, and assembled with the U.S. Department of Energy Systems Biology Knowledgebase (KBase) platform. The quality assessment of the raw data was conducted employing FastQC v0.12.1. The paired-end sequence was trimmed via Trimmomatic v0.36, where the trimming length was set to 20. Then, the sequence was assembled using SPAdes v3.15.3, a genome assembler for both regular and single-cell projects (8). The isolate was identified as *Pseudomonas aeruginosa* using the Type (Strain) Genome Server (https://tygs.dsmz.de/), with the closest reference genome being that of *Pseudomonas aeruginosa* DSM 50071 (GenBank accession number: FUXR00000000) (9). The strain was named MLSE01. The genome annotation was performed with NCBI PGAP employing the best-placed reference protein set (10). Default parameters were used for all software unless otherwise specified.

The length of *Pseudomonas aeruginosa* strain MLSE01 draft genome is 6,332,769 bp in 48 contigs with 47.47× coverage and 66.5% GC content. The annotation process identified 5,937 genes, including 5,821 protein-coding sequences. Key genomic features are summarized in Table 1.

**TABLE 1:** Genomic features of *Pseudomonas aeruginosa* MLSE01

Description of source	
Location	Kuakata, Bangladesh
Time	2024
Type	Seawater
Sequencing Summary	
Coverage	47.47
Number of raw reads	1,110,152
Total bases	543.3 M
*GC Content(%)*	67.8
Size	357.4 Mb
Assembly Report	
Tools used	SPAdes v. 3.15.3
Contigs	48
GC Content	66.5
Contig L50	4
Genome Length	6,332,769 bp
Contig N50	634,874 bp
Annotation Report	
Tool used	PGAP
Genes (total)	5,937
CDS (total)	5,869
Genes (coding)	5,821
CDSs (with protein)	5,821
rRNA	3, 1, 3 (5S, 16S, 23S)
tRNAs	57
ncRNAs	4
CDSs (without protein)	48

## Data Availability

The whole genome sequence is openly available in the NCBI (National Center for Biotechnology Information) Database under GenBank accession number JBLYEI000000000.1, BioProject PRJNA1230370. The unassembled raw sequence was submitted to the Sequence Read Archive (SRA) repository of NCBI under BioSample accession number SAMN47177160 and SRA accession number SRR36688095. The assembled genome has been submitted to BV-BRC and is available under genome ID 287.42017.
